# Disconnectome of the migraine brain: a “connectopathy” model

**DOI:** 10.1186/s10194-021-01315-6

**Published:** 2021-08-28

**Authors:** Marcello Silvestro, Alessandro Tessitore, Giuseppina Caiazzo, Fabrizio Scotto di Clemente, Francesca Trojsi, Mario Cirillo, Fabrizio Esposito, Gioacchino Tedeschi, Antonio Russo

**Affiliations:** 1 Headache Center, Department of Advanced Medical and Surgical Sciences, University of Campania “Luigi Vanvitelli”, Naples, Italy; 2 MRI Research Centre SUN-FISM, University of Campania “Luigi Vanvitelli”, Naples, Italy; 3 Institute for Diagnosis and Care ‘Hermitage-Capodimonte’, Naples, Italy

**Keywords:** Graph analysis, Migraine, Connectome, Advanced neuroimaging, Brain network

## Abstract

**Background:**

In the past decades a plethora of studies has been conducted to explore resting-state functional connectivity (RS-FC) of the brain networks in migraine with conflicting results probably due to the variability and susceptibility of signal fluctuations across the course of RS-FC scan. On the other hand, the structural substrates enabling the functional communications among the brain connectome, characterized by higher stability and reproducibility, have not been widely investigated in migraine by means of graph analysis approach. We hypothesize a rearrangement of the brain connectome with an increase of both strength and density of connections between cortical areas specifically involved in pain perception, processing and modulation in migraine patients. Moreover, such connectome rearrangement, inducing an imbalance between the competing parameters of network efficiency and segregation, may underpin a mismatch between energy resources and demand representing the neuronal correlate of the energetically dysfunctional migraine brain.

**Methods:**

We investigated, using diffusion-weighted MRI imaging tractography-based graph analysis, the graph-topological indices of the brain “connectome”, a set of grey matter regions (nodes) structurally connected by white matter paths (edges) in 94 patients with migraine without aura compared to 91 healthy controls.

**Results:**

We observed in migraine patients compared to healthy controls: i) higher local and global network efficiency (*p* < 0.001) and ii) higher local and global clustering coefficient (*p* < 0.001). Moreover, we found changes in the hubs topology in migraine patients with: i) posterior cingulate cortex and inferior parietal lobule (encompassing the so-called neurolimbic-pain network) assuming the hub role and ii) fronto-orbital cortex, involved in emotional aspects, and visual areas, involved in migraine pathophysiology, losing the hub role. Finally, we found higher connection (edges) probability between cortical nodes involved in pain perception and modulation as well as in cognitive and affective attribution of pain experiences, in migraine patients when compared to healthy controls (*p* < 0.001). No correlations were found between imaging and clinical parameters of disease severity.

**Conclusion:**

The imbalance between the need of investing resources to promote network efficiency and the need of minimizing the metabolic cost of wiring probably represents the mechanism underlying migraine patients’ susceptibility to triggers. Such changes in connectome topography suggest an intriguing pathophysiological model of migraine as brain “connectopathy”.

**Supplementary Information:**

The online version contains supplementary material available at 10.1186/s10194-021-01315-6.

## Introduction

In the last decades, driven by the ascendancy of network science [1], anatomically distributed components continuously sharing information with each other have been identified in the brain [2]. These functional connectivity patterns are normally organized as reproducible large-scale functional networks, called resting-state networks. More recently, structural neural substrates enabling functional communication have been explored by means of advanced diffusion-weighted MRI and tractography-based graph theory analysis, powerful tool to investigate the brain “connectome”, a set of grey matter regions (nodes) structurally connected by white matter paths (edges) [3]. Two distinct dimensions, along which brain connectome network is organized, ‘segregation’ and ‘integration’, provide a general framework that allows description and categorization of different disorders [4]. In particular, functional segregation is the ability for specialized processing to occur within densely interconnected nodes of brain regions, known as clusters or modules, while functional integration is the ability to rapidly combine specialized information from distributed brain regions.

In the past decade a plethora of studies has been conducted to explore resting-state functional connectivity (RS-FC) of the brain networks in migraine however conflicting results emerged due several issue such as low homogeneity of patient populations, different methodological approaches and the extreme variability and susceptibility of signal fluctuations across the course of RS-FC scan. On the other hand, the structural substrates enabling the functional communications among the brain network or connectome, characterized by high stability and reproducibility, have not been widely investigated in migraine [5].

Previous pivotal electroencephalographic, magnetoencephalographic and MRI observations, conducted in restricted cohorts of patients, showed a substantial reorganization of the brain connectome in migraine. More in-depth, several studies demonstrated an increased network segregation due to high structural and functional local connections among cortical nodes related to pain perception, processing and modulation [6–11]. However, increased network integration and connection density between hubs (also known as “rich club regions”) and nodes (also known as “non-rich club regions”) have been also reported in migraine without aura (MwoA) patients [10, 11].

We hypothesize that migraine patients may show a rearrangement of the brain connectome exhibiting increase both strength and density of connections, specifically affecting the nodes involved in multidimensional pain processing with a consequent reorganization of hubs topography. Due to the high energetic resources needed to promote network integration and segregation, we further hypothesize this pattern of brain connectome rearrangement may represent the neuronal correlate of the well-known energetic dysfunction of migraine brain.

Therefore, we conducted a whole-brain graph analysis to investigate the brain connectome using a probabilistic tractography-based evaluation in a large cohort of MwoA patients compared to healthy controls (HC).

## Materials and methods

### Study population and study design

One hundred episodic MwoA patients, according to the International Headache Society criteria (Headache Classification Subcommittee of the International Headache Society, 2013 and 2018) [12] were prospectively recruited between 2013 and 2019 from the migraine population referring to the Headache Center of the Department of Neurology at the University of Campania “Luigi Vanvitelli”. Demographic data were obtained as well as the following clinical features: age at migraine onset, disease duration, attacks frequency (day/month), aura duration, attacks pain intensity, assessed using visual analogic scale (VAS) (mean VAS score of migraine attacks experienced in the last month) and related disability (using Migraine Disability Assessment Scale -MIDAS and Headache Impact Test - HIT-6) (Table 1). Moreover, patients completed the Hamilton Depression Rating Scale (HDRS), Hamilton Anxiety Rating Scale (HARS). Pregnant women and patients with claustrophobia as well as comorbidities were excluded. All patients were both migraine-free and they were not taking medications for migraine attacks in the 3 days before and after scanning at least. Finally, patients were naïve for any commonly prescribed migraine preventive medications. Final analyses were conducted on 94 MwoA patients because six patients experienced migraine attacks within 3 days after the MRI scan.

**Table 1:** Demographic and clinical characteristics of patients with MwoA and HC

Parameter	Group	Mean ± SD	***P*** value
**Gender**	MwoA	23 M; 71F	0.08
	HC	33 M; 58F	
**Age (years)**	MwoA	30.5 ± 8.1	0.76
	HC	30.7 ± 8.4	
**Disease duration (years)**	MwoA	11.0 ± 7.7	–
**Frequency (migraine/month)**	MwoA	5.4 ± 3.3	–
**MIDAS**	MwoA	22.6 ± 13.8	–
**HIT-6**	MwoA	62.3 ± 6.8	–
**VAS of attack intensity**	MwoA	8.1 ± 1.0	–
**HARS**	MwoA	5.3 ± 0.9	–
**HDRS**	MwoA	5.7 ± 0.8	–

*MwoA* Migraine without aura, *HC* Healthy controls, *M* Male, *F* Female, *MIDAS* Migraine disability assessment scale, *HIT-6* Headache impact test-6, *VAS* Visual analogic scale, *HARS* Hamilton anxiety rating scale, *HDRS* Hamilton depression rating scale

Ninety-one age and sex-matched subjects with less than a few spontaneous non-throbbing headaches per year, with no history of migraine, pregnancy, claustrophobia as well as other comorbidities were recruited as HC via advertisements placed in the hospital (e.g. posters and flyers), word-of-mouth referrals and from a database of research volunteers of the MRI Research Centre of the University Hospital of Naples.

### Standard protocol approvals, registrations, and patient consents

The study was approved by the Ethics Committee of University of Campania “Luigi Vanvitelli”, and written informed consent was obtained from all subjects according to the Declaration of Helsinki.

### Imaging parameters

Magnetic resonance images were acquired on a General Electric 3-Tesla MRI scanner equipped with an 8channel parallel-head coil. Three-dimensional T1-weighted sagittal images were acquired with a gradient-echo sequence IR-FSPGR (TR = 6988 ms, TI = 1100 ms, TE = 3.9 ms, flipangle = 10, voxel size = 1 mm × 1 mm × 1.2 mm). Whole-brain diffusion-weighted MRI was performed by using a spin-echo echo-planar imaging (EPI) sequence (repetition time = 10,000 ms, echo time = 83.2 ms, field of view = 320 mm, isotropic resolution = 2.5 mm, b value = 1000 s/mm, 32 isotropically distributed gradient directions, frequency encoding left-right - LR).

### Data pre-processing

Motion, eddy currents correction and brain tissue extraction (BET) of diffusion-weighted images were performed using FSL version 5.0.8 [13]. The distribution of fiber orientations at each voxel was estimated, producing all diffusion orientation maps for probabilistic tractography, with Bedpostx, a command line tool of the FSL package [14]. After co-registration of dMRI images with T1-weighted images, a cortical grey matter parcellation was performed using the Automated Anatomical Labeling Atlas (AAL) which includes 90 cortical and subcortical regions [15]. The obtained structures were afterward used as ROIs for fibre tracking. Probabilistic fibre tracking was performed in FSL according to Behrens and colleagues [16]. Probabilistic tractography was applied by sampling_5000 streamline fibres per each voxel to estimate the connection probability. For each sampled fibre, a sample direction was first drawn from the local direction distribution at the seed voxel, then a new sample direction, from the local distribution, was obtained at the next position, located 0.5 mm along the previous direction, etc. For each seed region, 5000 × n fibres were sampled (“n” represents the number of voxels in the region). The number of fibres passing through a given region divided by 5000 × n is finally given as the connection probability from the seed region to the target region [17]. In the present study, each cortical region was selected as the seed region and its connection probability to each of the other 90 regions was calculated [17].

### Network construction and graph-theoretic measures

From the tractography results, each data set was transformed into a connectivity matrix, measuring connection probability from the seed region to the target region. Each individual network is thus represented by a symmetric 90 × 90 matrix, in which each row and column represents a node and each element represents an edge. The raw individual networks are likely to contain spurious connections due to noise and algorithm errors; however, the graphs can be controlled for spurious connections using group-level non-parametric statistics [18]. The non-parametric sign test was applied by taking each individual as a sampling point, with the null hypothesis being that there is no existing connection (i.e., connectivity weight = 0). The Bonferroni method was used to correct for multiple comparisons across all node pairs within the network. For each group data set (MwoA and HC), a corrected *p* < 0.05 was deemed to have a connection with the node pair surviving. As a result, a binary matrix (1 for node pairs with a connection and 0 for node pairs without a connection) was generated for each group of WM networks. This binary mask was then applied to each individual subject network to remove the spurious connections [19]. In this way, for all estimated white matter (WM) networks, the network density, which is the fraction of remaining connections to all possible connections, becomes equalized across all subjects of the cohorts, enabling a proper comparative analysis of network topological features between the groups [19]. We calculated the following network measures [20] with the Brain Connectivity:

Measures of network integration:

- Characteristic path length: is the minimum number of edges that must be traversed to go from one node to another and is the most commonly used measure of functional integration;
- Global efficiency: reflects the capacity for network-wide communication. Efficiency is inversely related to path length but is numerically easier to use to estimate topological distances between elements of disconnected graphs.

Measures of network segregation:

- Clustering coefficient: estimates the fraction of triangles around an individual node and is equivalent to the fraction of the node’s neighbours that are also neighbours of each other;
- Modularity: quantifies the degree to which a network may be subdivided into such clearly delineated and non-overlapping groups of nodes, the so-called modules.

Measures of node centrality:

- Node degree: quantifies the number of connections that link a node to the rest of the network. This is a fundamental network measure since most other measures are ultimately linked to node degree;
- Node strength: is the sum of weights of links connected to the node;
- Betweenness centrality: is the fraction of all shortest paths in the network that contain a given node. Nodes with high values of betweenness centrality participate in a large number of shortest paths. Betweenness centrality is a widely used measure to identify the most central nodes in a graph (the so-called hubs), which are associated to those nodes that acts as bridges between the others nodes [21]. In the present work, the mean betweenness centrality was calculated for each node in MwoA and HC groups separately. Subsequently, regions on the topographic betweenness centrality map with values in the 80th percentile were defined as group hub regions [22, 23].
- Eigenvector centrality: is a measure of the influence of a node in a network. In particular, it estimates the connection between nodes with high centrality. A high eigenvector score means that a node is connected to many nodes who themselves have high scores of centrality.

### Statistical analysis

The network-based statistics (NBS) tool is a MATLAB toolbox for testing hypotheses about the human connectome [24]. It was used to measure a between-group difference using intensity of network connectivity values comprising pairs of regions with FDR correction for multiple comparisons. The analyses has been corrected for the age and sex of participants. For each nodal and global measures, the null hypothesis MwoA = HC was tested using a t-test and was rejected at *p* < 0.05 for all network measures considered and false discovery rate for multiple hypothesis testing correction for all network nodes. The software STATA version 14 was used for the analysis of demographic and clinical data and for the correlation between clinical and imaging data. The comparison between MwoA patients and HC on demographic aspects and clinical severity parameters of disease was performed by means of t-test and Chi-squared, as appropriate. *p* < 0.05 was considered statistically significant. Within the sample of MwoA patients, the correlation analysis between the imaging (efficiency, path length, clustering coefficient, modularity and node strength) and clinical parameters of disease severity was carried out by means of Spearman’s rank correlation coefficient. The value of *p* < 0.05 was considered statistically significant; the Bonferroni correction for multiple comparisons was applied.

### Data availability statement

Data requests can be directed to author (antonio.russo@unicampania.it).

## Results

There was no significant difference between the two groups (94 patients with MwoA and 91 HC) regarding age and male/female ratio. Demographic and baseline headache characteristics of patients included in the study are reported in Table 1.

**Fig. 1:**
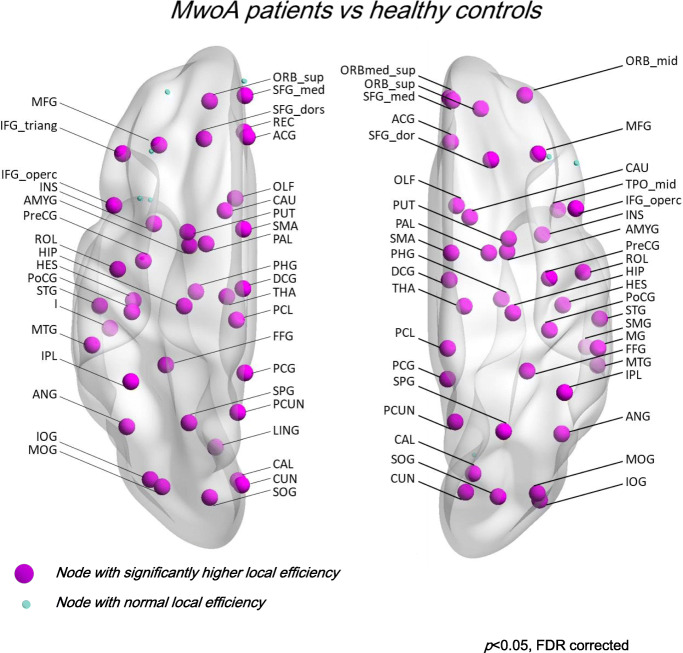
Representation of nodes with significantly higher local efficiency (*t* score) in MwoA patients compared to HC data sets (*p* < 0.05 corrected for multiple comparison)

**Fig. 2:**
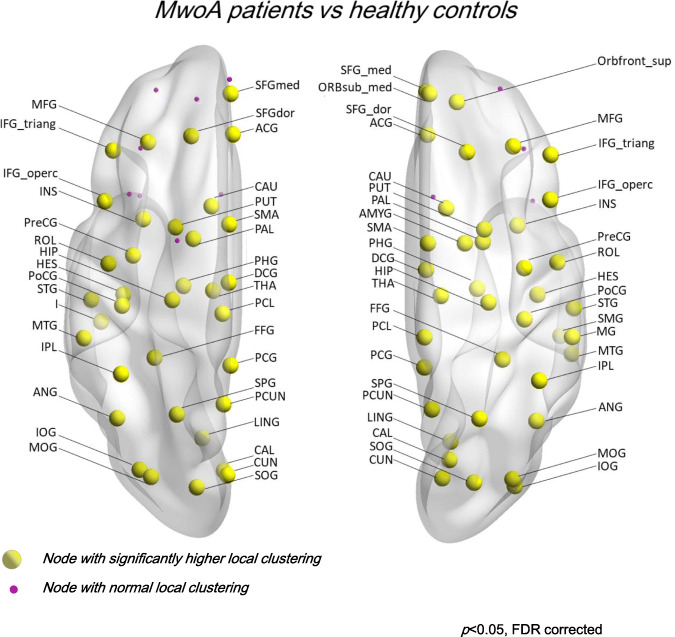
Representation of nodes with significantly higher local clustering (*t* score) in MwoA patients compared to HC data sets (*p* < 0.05 corrected for multiple comparison)

MwoA patients compared to HC showed:

1. At the level of network integration, higher local and global efficiency (MwoA vs HC *p* < 0.001) without differences in local or global path length (see Fig. 1 and supplementary material 1).
2. At the level of network segregation, higher local and global clustering coefficient (MwoA vs HC *p* < 0.001) without differences in modularity (see Fig. 2 and supplementary material 2).
3. At the level of nodes centrality, higher local and global node strength, and eigenvector centrality in numerous nodes and lower betweenness centrality in numerous nodes (*p* < 0.05, FDR corrected) (See supplementary materials 1 and 2).

**Fig. 3:**
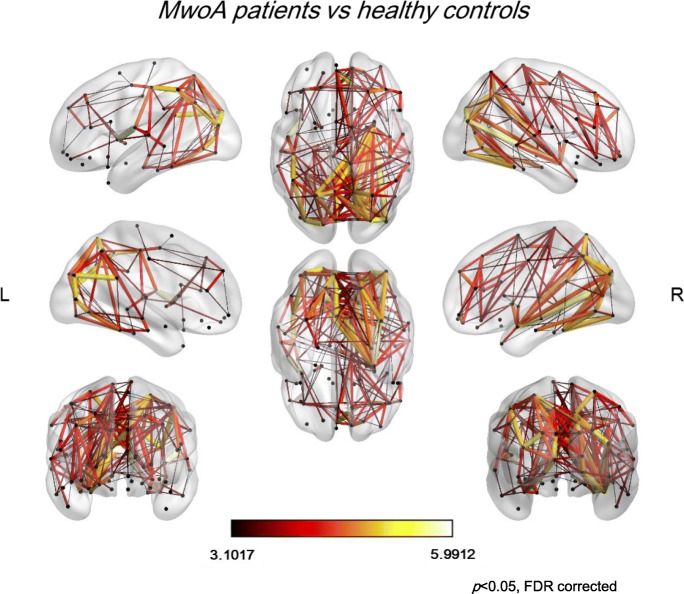
Representation of nodes and edges with significantly higher connection probability (*t* score) in MwoA patients compared to HC data sets (*p* < 0.05 corrected for multiple comparison)

**Fig. 4:**
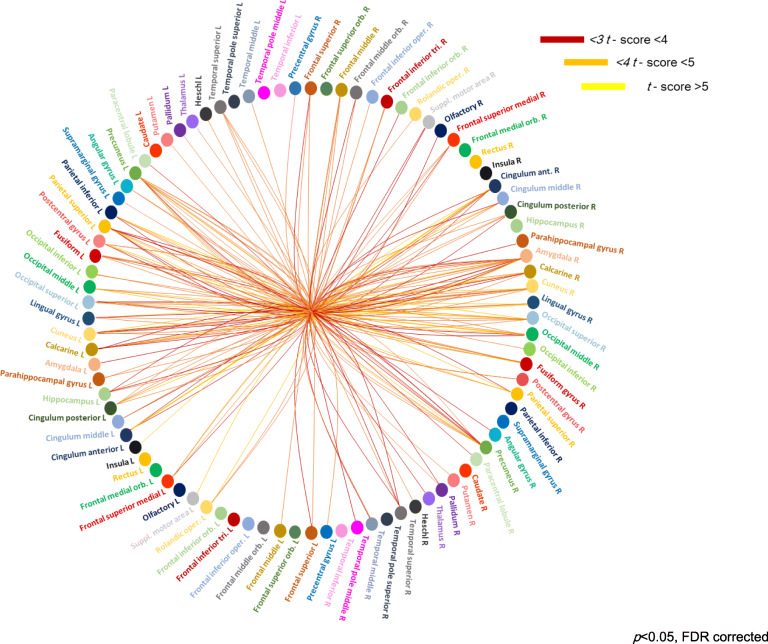
Connectogram of nodes and edges showing significantly higher connection probability (*t* score) in precuneus, cuneus, amygdala, calcarine cortex, posterior cingulate cortex, anterior cingulate cortex, postcentral gyrus, superior parietal lobule, lingual and fusiform gyri, middle frontal gyrus and inferior and superior parietal lobules in MwoA patients compared to HC data sets (*p* < 0.05 corrected for multiple comparison)

The NBS analysis revealed a significantly higher connection probability in MwoA patients when compared to HC, respectively in 118 pairs of nodes (*p* = 0.0002) (see Figs. 3 and 4).

**Fig. 5:**
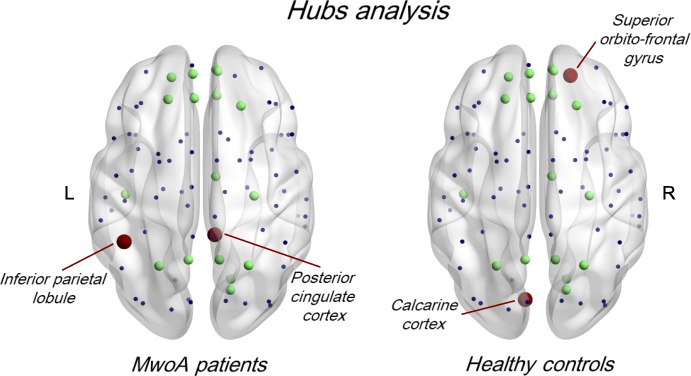
Hubs regions with groups betweenness centrality scores in the 80th percentile displayed on brain on MNI template surface. Red spots: hubs exclusively in MwoA patients or HC acquisition schemes. Green spots: common hubs in MwoA patients and HC acquisition schemes. Blue dots: common nodes in MwoA patients and HC acquisition schemes

The distribution of hubs across the whole connectome was similar between data-sets derived from two groups, except for the right posterior cingulate cortex (PCC) and left inferior parietal lobule (IPL) (based on the AAL atlas) identified as hubs only in MwoA patients-derived matrices. Similarly, the right orbitofrontal cortex (OFC) and left calcarine cortex (based on the AAL atlas) were identified as hubs in HC-derived but not in MwoA patients-derived matrices (Fig. 5).

Finally, no correlations have been found between connectome measures and clinical parameters of disease severity.

## Discussion

According to graph theory, a network (or a graph) is defined as a set of discrete elements, referred to as nodes or vertices, and their mutual relationships, the so-called edges or links, which can be summarized in the form of a connection matrix [25, 26].

Network/graph topology can be quantitatively described by means of several global indices (derived from averages of local indices) assessing the level of network integration (e.g. efficiency and path length), segregation (e.g. clustering and modularity) and nodes centrality (edges assessment, betweenness centrality, node strength and eigenvector centrality) [20]. Integration and segregation, two mutually dependent principles of connectome arrangement, are competing dimensions conditioning the overall healthy brain function, according to their reciprocal balance (the so-called ‘pareto front’) [25, 26].

Herein, we demonstrated (at the level of network integration) increased brain network global efficiency, but not global path length changes, and (at the level of network segregation) higher mean global clustering, but not modularity, in MwoA patients when compared to HC.

Moreover, findings of nodes centrality showed an overall increase in the values of connection probability by edges, across numerous pairs of anatomical regions, and identified as hubs right PCC and left IPL only in MwoA patients and left calcarine cortex and right OFC only in HC, when comparing the two groups data sets. Finally, no significant correlations between connectome data and clinical parameters of migraine burden have been found.

The global efficiency is a relevant index of the network ability to withstand and manage large flow of information. On the other hand, the path length estimates the minimum number of edges that must be traversed to go from one node to another and, therefore, together with the path density, it is expected to be inversely correlated to the network efficiency. Importantly, an increased global efficiency does not mean better functioning, considering that i) mammals brain is usually characterized by a lower global efficiency (and a higher local efficiency) compared to a model of random network, and ii) higher global integration has been associated with a higher general risk factor for brain diseases [27, 28]. In particular, the developmental “local-to-global” reorganization of brain connectivity during adolescence represents a factor of high vulnerability for the occurrence of neuropsychiatric disorders (e.g. psychosis, mood disorders and depression). Interestingly, the adolescence is also the period in which more frequently migraine arises.

The clustering coefficient is used to establish the sets of adjacent strongly interconnected nodes that affects the network connectivity [29]. The overall higher clustering, observed in our sample of MwoA patients compared with HC, characterizes complex networks and is associated with both increased local efficiency of information transfer and network robustness. This finding is in line with previous pivotal electroencephalographic, magnetoencephalographic and MRI observations that showed an increased clustering coefficient in a restricted cohort of migraine patients, due to increased structural and functional local connections among pain-related brain regions inducing a substantial reorganization of cortical networks [6–11]. It is noteworthy that increased clustering without significant changes in the path length (e.g. shortest absolute path length) suggests a disrupted cortical topological arrangement with a break in the hierarchical structure of brain connectome [6–11]. Likewise, increased global efficiency and connection density between hubs (also known as “rich club regions”) and other nodes (also known as “non-rich club regions”) have been demonstrated in MwoA patients, suggesting a complex and pervasive dysfunction in migraine [10, 11].

The analysis of nodes centrality aimed to investigate the most probable connections (edges) between nodes and to identify the nodes more probably involved by the connections themselves (hubs), and, therefore, this parameter allows exploring the influence of edges and hubs on the connectome topology. In particular, a significantly higher occurrence of cortical areas known to be involved in pain perception and modulation, in cognitive and affective attribution of pain experiences (e.g. anterior cingulate cortex, postcentral gyrus, superior parietal lobule, IPL, PCC, precuneus, cuneus, amygdala and middle frontal gyrus) and in visual processing (lingual and fusiform gyri and calcarine cortex) has been found among the abnormal edges in MwoA patients [30–33]. This finding is in line with a recent observation reporting higher number of connections (streamlines) involving several cortical regions (e.g. superior frontal gyrus, precentral and postcentral giri) when comparing both episodic and chronic MwoA patients with HC [34].

Furthermore, we found that several nodes, such as right PCC and left IPL, that are notoriously part of the so called neuro-limbic pain network, due to their high degree or centrality, are found as hubs in migraine patients but not in HC [30]. On the other side, we found that several hubs, such as right OFC and left calcarine cortex, due to a reduced betweenness centrality, lose their hub roles and work as “simple” nodes in migraine patients. It is known that the specialized roles played by hubs in integrative processing are essential in maintaining brain network healthiness as a whole and, therefore, both the presence of supernumerary hubs and their absence may be implicated in the pathophysiology of brain disorders [31–33].

Altogether, our findings, as widely supported by converging evidences [6–9, 34, 35], demonstrate that migraine brain may be characterized by a specific connectome disconnectivity or disconnectome caused by an imbalance between the two major competing principles that handle the normal connectome organization: i) the need to invest resources to promote network efficiency and segregation and ii) the need to minimize the physical and metabolic cost of wiring [25]. More specifically, the increased efficiency, clustering and strength of both nodes and their connections observed in migraine patients underpin a non-linear increase in energy demand to manage the large flow of internal and external inputs, justifying increased energy requirements and greater brain vulnerability to stressors. Furthermore, the hubs represent hot spots of both vulnerability and energy demand, due to specific local physiology [36]. In this frame, as previously supposed, the mismatch between energy resources and demand may be able to ignite the major alarm system of the brain, the trigemino-vascular system, leading to migraine attack and consequent sickness behavior to restore brain energy metabolism [9, 35]. According to the interpretation of migraine in evolutionary perspective [37, 38], migraine could be one of the evolutionary prices that human species has to pay for developing a highly-connected brain and, in turns, a highly performing central nervous system. Probably, over time, inadequate or suboptimal nutritional and environmental changes might have turned a Darwinian advantage into a disabling disadvantage.

We believe that the absence of significant correlations between graph-theoretical parameters and clinical measures of disease severity could suggest the innate characteristic of observed findings independently from the clinical phenotype that will developed in the course of disease. On the other hand, we cannot exclude that the functional and structural connectome rearrangement could be the result of remodelling processes induced by repetitive migraine attacks, thereby interpreting migraine as a disease model of allostatic load [39]. Indeed, we found that several nodes increased their centrality playing as hubs, while several hubs reduced it, working as simple nodes. Among the first, we found the PCC (involved in the balance between internally and externally focused attention and engaged in both pain perception and multisensory integration) and the IPL (notoriously involved in the sensory-discriminative aspects of painful stimuli such as quality, intensity, spatial and temporal features as well as in the affective and cognitive components of pain such as pain unpleasantness) [40]. However, the reduced centrality of the OFC and, in turns, the reduced ability in working as hub and managing a high flow of inputs may partly contribute in elucidating the close relationship between the difficulties of migraine patients in coping with deeper emotional aspects and migraine phenomenon [41, 42]. Indeed, the OFC plays its critical role in the dynamic filtration of emotional stimuli and emotional decisions, relying on the overview of both external and internal environmental factors, to such an extent that a *dynamic filtering* theory has been proposed as a neural mechanism to account for the role of the OFC in emotional regulation [42]. Likewise, the lack of the calcarine hub within the connectome arrangement in MwoA patients may underpin the well-known role of visual network abnormalities in mechanisms underlying migraine pathophysiology [32, 36].

We are aware that our study has some strengths but is not exempt from some limitations as well. Among the first, there are the high sample size and homogeneity, which protects from confounding results conditioned by selection bias and ensures on the results reproducibility. Similarly, patients enrolled were “drug naïve” in order to exclude a putative pharmacological effects on connectome organization. On the other hand, the gender matching between patients and controls was not ideal for this type of study in the present study and we employed connectome global metrics despite there are not converging opinions about their specificity and there is a lot of evidence suggesting a gender bias on brain connectivity [43–47]. Nevertheless, to overcome this bias, we balanced the global measures with further in-depth local centrality investigations, aimed to improve the identification of the most probable edges and hubs. Moreover, we investigated migraine interictal period not considering the temporal distance with the last or the next migraine attack. However, based on the well-known stability of structural connectome compared to fleeting functional connectome fluctuations, we believe that our findings could be not influenced by the distance from migraine attacks [28].

## Conclusion

In conclusion, we suggest a new stimulating pathophysiological interpretation model of migraine as a “connectopathy”. Future prospective advanced neuroimaging studies remain of paramount importance to further clarify whether observed structural disconnectivity could represent an innate characteristic of migraine brain or the result of a remodelling process induced by the experience of repetitive migraine attacks.

## Supplementary Information

**Additional file 1: Supplementary table 1.** Statistically significant differences in local connectome measures (t-test) in patients with MwoA compared to HC (local efficiency, clustering coefficient and node strength local values are higher in patients with MwoA compared to HC, betweenness centrality values are lower in patients with MwoA compared to HC, eigenvector centrality values are higher in patients with MwoA compared to HC except for marked nodes*).

**Additional file 2: Supplementary table 2.** Statistically significant differences in global connectome measures (mean values and t-test) in patients with MwoA compared to HC.

## Data Availability

Data requests can be directed to author (antonio.russo@unicampania.it).

## Glossary

AAL: Anatomical Labelling Atlas
BET: Brain tissue extraction
CSA: Constant solid angle
DTI: Diffusion Tensor Imaging
EPI: Echo-planar imaging
FDR: False discovery rate
GFA: Generalized fractional anisotropy
HARS: Hamilton Anxiety Rating Scale
HC: Healthy controls
HDRS: Hamilton Depression Rating Scale
HIT-6: Headache Impact Test − 6
IPL: Inferior parietal lobule
MIDAS: Migraine Disability Assessment Scale
MwoA: Migraine without aura
NBS: Network-based statistic
OFC: Orbitofrontal cortex
PCC: Posterior cingulate cortex
RS-fMRI: Resting-state-functional MRI
VAS: Visual analogic scale
WM: White matter
