# Supplementary material for: Disconnectome of the migraine brain: a “connectopathy” model
Source: J Headache Pain. 2021 Aug 28;22(1):102. doi: 10.1186/s10194-021-01315-6 (PMC8400754; doi:10.1186/s10194-021-01315-6)
Supplement: Supplementary file 1 — Additional file 1: Supplementary table 1. Statistically significant differences in local connectome measures (t-test) in patients with MwoA compared to HC (local efficiency, clustering coefficient and node strength local values are higher in patients with MwoA compared to HC, betweenness centrality values are lower in patients with MwoA compared to HC, eigenvector centrality values are higher in patients with MwoA compared to HC except for marked nodes*). [file 10194_2021_1315_MOESM1_ESM.docx]

**Supplementary table_1**Statistically significant differences in local connectome measures (t-test) in patients with MwoA compared to HC (local efficiency, clustering coefficient and node strength local values are higher in patients with MwoA compared to HC, betweenness centrality values are lower in patients with MwoA compared to HC, eigenvector centrality values are higher in patients with MwoA compared to HC except for marked nodes*)

|  | **Efficiency** | **Clustering coefficient** | **Node strength** | **Eigenvector centrality** | **Betweenness centrality** |
| --- | --- | --- | --- | --- | --- |
| **Precentral_L** | 0.000003 | 0.000006 | 0.00005 | - | - |
| **Precentral_R** | 0.000028 | 0.000074 | 0.00001 | - | 0.002054 |
| **Frontal_Sup_L** | 0.000038 | 0.000059 | 0.00001 | - | - |
| **Frontal_Sup_R** | 0.000068 | 0.000098 | 0.000001 | 0.017048* | 0.004473 |
| **Frontal_Sup_Orb_L** | 0.031409 | - | - | 0.000586* | 0.000001 |
| **Frontal_Sup_Orb_R** | 0.027265 | 0.034164 | 0.02299 | 0.000467* | 0.000053 |
| **Frontal_Mid_L** | 0.000033 | 0.000042 | 0.00001 | - | - |
| **Frontal_Mid_R** | 0.000008 | 0.000031 | 0.000001 | 0.021077* | - |
| **Frontal_Mid_Orb_L** | - | - | 0.0044 | 0.001975* | - |
| **Frontal_Mid_Orb_R** | 0.030019 | - | 0.0087 | 0.003892* | - |
| **Frontal_Inf_Oper_L** | 0.000846 | 0.001546 | 0.0182 | - | 0.004496 |
| **Frontal_Inf_Oper_R** | 0.012249 | 0.016787 | 0.0085 | 0.007425* | - |
| **Frontal_Inf_Tri_L** | 0.016334 | 0.022236 | - | - | - |
| **Frontal_Inf_Tri_R** | - | - | - | 0.000889* | - |
| **Frontal_Inf_Orb_L** | - | - | - | 0.001740* | 0.000001 |
| **Frontal_Inf_Orb_R** | - | - | - | 0.00006* | 0.000001 |
| **Rolandic_Oper_L** | 0.000586 | 0.001875 | 0.0025 | - | 0.006947 |
| **Rolandic_Oper_R** | 0.020280 | 0.029353 | 0.0193 | 0.0118154* | 0.005338 |
| **Supp_Motor_Area_L** | 0.000034 | 0.000047 | <0.0001 | - | - |
| **Supp_Motor_Area_R** | 0.000002 | 0.000003 | <0.0001 | - | - |
| **Olfactory_L** | 0.003580 | 0.008908 | - | 0.000220* | - |
| **Olfactory_R** | 0.013700 | 0.032335 | - | 0.000431* | 0.006939 |
| **Frontal_Sup_Medial_L** | 0.002349 | 0.001833 | 0.0009 | - | - |
| **Frontal_Sup_Medial_R** | 0.001685 | 0.001347 | 0.0001 | - | - |
| **Frontal_Med_Orb_L** | - | - | - | 0.000613* | - |
| **Frontal_Med_Orb_R** | 0.017300 | 0.012051 | 0.0066 | 0.000383* | - |
| **Rectus_L** | 0.007691 | 0.007104 | - | 0.000106* | 0.000039 |
| **Rectus_R** | - | - | - | <0.00001* | 0.000115 |
| **Insula_L** | 0.000162 | 0.000751 | 0.0001 | - | - |
| **Insula_R** | 0.011948 | 0.031328 | 0.0030 | 0.000621* | 0.000045 |
| **Cingulum_Ant_L** | 0.000253 | 0.000314 | <0.0001 | - | - |
| **Cingulum_Ant_R** | 0.000024 | 0.000027 | <0.0001 | - | - |
| **Cingulum_Mid_L** | 0.000006 | 0.000007 | <0.0001 | - | - |
| **Cingulum_Mid_R** | 0.000004 | 0.000005 | <0.0001 | - | - |
| **Cingulum_Post_L** | 0.000011 | 0.000016 | <0.0001 | 0.002598 | - |
| **Cingulum_Post_R** | 0.000010 | 0.000013 | <0.0001 | 0.002868 | - |
| **Hippocampus_L** | 0.003925 | 0.006242 | 0.0078 | - | - |
| **Hippocampus_R** | 0.018781 | 0.027160 | 0.0339 | - | - |
| **Para_Hippocampal_L** | 0.018267 | 0.021635 | 0.0047 | - | - |
| **Para_Hippocampal_R** | 0.019578 | 0.031476 | 0.0127 | - | - |
| **Amygdala_L** | 0.016412 | 0.019133 | - | - | - |
| **Amygdala_R** | 0.000227 | 0.001222 | 0.0078 | - | - |
| **Calcarine_L** | 0.000106 | 0.000043 | <0.0001 | 0.001336 | - |
| **Calcarine_R** | 0.000707 | 0.000192 | <0.0001 | 0.000348 | 0.000904 |
| **Cuneus_L** | 0.000001 | 0.000001 | <0.0001 | 0.009032 | - |
| **Cuneus_R** | 0.000003 | 0.000002 | <0.0001 | 0.000309 | - |
| **Lingual_L** | 0.000370 | 0.000436 | <0.0001 | 0.002660 | - |
| **Lingual_R** | - | 0.028088 | <0.0001 | 0.001643 | 0.001107 |
| **Occipital_Sup_L** | 0.000021 | 0.000011 | <0.0001 | 0.009190 | - |
| **Occipital_Sup_R** | 0.000088 | 0.000050 | <0.0001 | <0.00001 | - |
| **Occipital_Mid_L** | 0.001213 | 0.001028 | - | - | - |
| **Occipital_Mid_R** | 0.000020 | 0.000030 | <0.0001 | <0.00001 | - |
| **Occipital_Inf_L** | 0.003022 | 0.002247 | - | - | - |
| **Occipital_Inf_R** | 0.040596 | - | - | - | - |
| **Fusiform_L** | 0.001597 | 0.002003 | - | 0.018142 | - |
| **Fusiform_R** | 0.020524 | 0.019665 | - | 0.018577 | - |
| **Postcentral_L** | 0.000004 | 0.000007 | <0.0001 | - | - |
| **Postcentral_R** | 0.000343 | 0.000371 | <0.0001 | - | - |
| **Parietal_Sup_L** | 0.000011 | 0.000013 | <0.0001 | 0.008834 | - |
| **Parietal_Sup_R** | 0.000064 | 0.000072 | <0.0005 | 0.003329 | - |
| **Parietal_Inf_L** | 0.000675 | 0.000707 | - | - | - |
| **Parietal_Inf_R** | 0.009382 | 0.011517 | - | - | - |
| **Supramarginal_L** | 0.002632 | 0.002489 | 0.0008 | - | - |
| **Supramarginal_R** | 0.004281 | 0.007214 | - | - | - |
| **Angular_L** | 0.003532 | 0.003164 | <0.0001 | - | - |
| **Angular_R** | 0,001353 | 0.001802 | - | 0.009720 | - |
| **Precuneus_L** | 0,000002 | 0.000004 | <0.0001 | 0.005616 | - |
| **Precuneus_R** | 0,000000 | 0.000001 | <0.0001 | 0.000340 | - |
| **Paracentral_Lobule_L** | 0,000005 | 0.000007 | <0.0001 | - | - |
| **Paracentral_Lobule_R** | 0,000001 | 0.000000 | <0.0001 | - | - |
| **Caudate_L** | 0.003800 | 0.007673 | 0.0033 | 0.001254* | - |
| **Caudate_R** | 0.005179 | 0.009219 | 0.0004 | 0.015925* | - |
| **Putamen_L** | 0.000763 | 0.001810 | 0.0110 | - | - |
| **Putamen_R** | 0.006560 | 0.013653 | 0.0002 | 0.006921* | - |
| **Pallidum_L** | 0.000372 | 0.000831 | - | - | - |
| **Pallidum_R** | 0.008449 | 0.010690 | - | 0.013360* | - |
| **Thalamus_L** | 0.000440 | 0.001066 | - | - | - |
| **Thalamus_R** | 0.001511 | 0.003964 | 0.0057 | - | - |
| **Heschl_L** | 0.038768 | 0.098420 | - | - | - |
| **Heschl_R** | 0.001180 | 0.001120 | 0.0016 | 0.012743* | - |
| **Temporal_Sup_L** | 0.001079 | 0.001965 | <0.0001 | - | 0.002496 |
| **Temporal_Sup_R** | 0.000607 | 0.002263 | <0.0001 | - | 0.0000001 |
| **Temporal_Pole_Sup_L** |  | - | - | 0.002802* | - |
| **Temporal_Pole_Sup_R** |  | - | - | 0.000528* | - |
| **Temporal_Mid_L** | 0.003442 | 0.005267 | - | - | - |
| **Temporal_Mid_R** | 0.015359 | 0.038818 | - | - | - |
| **Temporal_Pole_Mid_L** |  | - | - | - | - |
| **Temporal_Pole_Mid_R** | 0.013329 | 0.020627 | - | - | - |
| **Temporal_Inf_L** | 0.000678 | 0.001536 | - | - | - |
| **Temporal_Inf_R** | 0.022629 | - | - | - | - |
