# Supplementary material for: Disconnectome of the migraine brain: a “connectopathy” model
Source: J Headache Pain. 2021 Aug 28;22(1):102. doi: 10.1186/s10194-021-01315-6 (PMC8400754; doi:10.1186/s10194-021-01315-6)
Supplement: Supplementary file 2 — Additional file 2: Supplementary table 2. Statistically significant differences in global connectome measures (mean values and t-test) in patients with MwoA compared to HC. [file 10194_2021_1315_MOESM2_ESM.docx]

**Supplementary table_2**Statistically significant differences in global connectome measures (mean values and t-test) in patients with MwoA compared to HC

|  | **Efficiency** | **Path length** | **Clustering coefficient** | **Modularity** | **Node strength** |
| --- | --- | --- | --- | --- | --- |
| **MwoA (mean value)** | 0.0349 | 54.5883 | 0.0024 | 0.4817 | 0.9709 |
| **HC (mean value)** | 0.0406 | 40.7106 | 0.0028 | 0.4816 | 1.1353 |
| **MwoA vs HC t-test (p value)** | **0.001** | 0.3512 | **<0.001** | 0.9728 | **0.001** |
